# “Helping to Bridge the Gap Between Genetics and Development:ˮ Julian Huxley, Early 20th Century Oxford Biology, and the Epigenetic Origins of Animal Characters

**DOI:** 10.1007/s10739-025-09829-4

**Published:** 2025-10-03

**Authors:** Stefan Bernhardt-Radu

**Affiliations:** University of Leeds, Leeds, UK

## Abstract

Julian Huxley is remembered as the author of his landmark 1942 *Evolution: The Modern Synthesis.* Nowadays, however, he is criticized for having reduced biology to the selection of genes. Some have nevertheless suggested that Huxley’s biological views were more expansive—including rather than excluding issues regarding development or environment. In this paper, using hitherto unexamined sources, I show that Huxley’s developmental understanding of animal characters was rooted in his education at Oxford in the early 20th century. From embryologically and physiologically trained Oxford teachers, he learned to see characters as things that could not be predicted from the cell’s physico-chemical properties. Characters arose anew through dynamic interactions between parts. Huxley and his teachers labeled these as “epigenetic” processes that integrated multiple cross-pollinating causes such as heredity and development. After briefly exploring Huxley’s understanding of character development, I show how we can get to grips with Huxley’s biological views by exploring the context of his education at Oxford from 1906 to 1909. I then show how Huxley received and used these ideas, before I illustrate how they played an important role in his academic and socio-political work.

*Evolution: The Modern Synthesis* is Julian Huxley’s most famous book, re-printed as recently as 2009 (Huxley 1942; Huxley et al. 2009). Nevertheless, it is today the subject of criticism for having neglected development in biology and evolution. It is said that the book sees individual development as “of no use for the understanding of evolutionary diversification. Development was all that follows from the genetic information,” having no creative role itself (Esposito 2013, p. 51; see also Walsh 2015; Peterson 2016, p. 10; Nicoglou 2024, p. 100). This is not a universal opinion. The authors of the foreword to the 2009 edition of Huxley’s book have noted his concern for the “developmental effects of genes and of the ontogenetic process in general” (Huxley et al. 2009, p. 5; see also Baedke 2024; Waters and Van Helden 1992). More broadly, this underscores the point that, as Joe Cain has argued, there was more than one synthesis made by Huxley in his lifetime (Cain 2010, p. 360). According to Vassiliki Betty Smocovitis, however, Huxley is “the most underappreciated if not misunderstood figure” among the synthesizers (Smocovitis 2019, p. 32). Though Huxley has been elevated in the 2009 republication of his 1942 book, the problem remains that “too little of a substantive historical nature is stated about Huxley’s own version of the modern synthesis and little or no attempt is made to historicize or contextualize it” (Smocovitis 2023, p. 40). This paper historicizes one theme of Huxley’s biological thinking that made it into his 1942 book: his understanding of animal characters. I show that Huxley’s view of animal characters, which privileged neither genetics nor development, can be understood when traced back to his education at Oxford in 1906–1909.

Huxley’s biological views have already been shown to be more expansive. C. Kenneth Waters has warned that we should “resist the temptation to define [Huxley’s] scientific practice purely in terms of his interest in evolutionary biology” (Waters 1992, p. 21; Lamm 2011; see also Baedke 2024). Huxley made it evident in his 1942 book that his concern was to see the study of genes and development as complementary. In the *Preface* to his 1942 book, Huxley wrote that “any originality which this book may possess lies partly in its attempting to generalize [Fisher’s ideas regarding changes of mutation-effects] still further, by stressing the fact that a study of the effects of genes during development is as essential for an understanding of evolution as are the study of mutation and that of selection” (Huxley 1942, p. 8). Development and genetics were equally important to understand the inconstant “effects of genes,ˮ and, more broadly, to grasp the origins of animal characters.

But a “Mendelian Revolution” may obscure Huxley’s concern with development (Bowler 1989). The traditional story, partly crafted by Huxley himself, is that the arrival of genetics allowed the revival of Darwinism in a new synthetical form around the 1930s (Huxley 1942; Bowler 1989; Bashford 2022). In their edited collection on Huxley, Waters and Albert Van Helden have suggested that this story distorts our understanding of his work. Waters, with cues from historian Frederick Churchill, has proposed that Huxley aimed to develop an “embryological synthesis” (Waters 1992, p. 21). Churchill has focused in more depth on Huxley’s embryological work, particularly his and De Beer’s *The Elements of Experimental Embryology* (Churchill 1992, p. 108). Olby briefly mentioned that Huxley, along with De Beer, aimed to give a “developmental account of gene action” (Olby 1992, p. 66). Neither asked why Huxley aimed to bridge genetics and development in the first place. A. Jan Witkowski, following John Baker’s suggestion that Huxley, in his laboratory work, “was concerned with ontogeny,” argued that we should consider Huxley’s Oxford tutors: John Wilfred Jenkinson and Geoffrey Smith. He quoted Baker saying that “one can scarcely doubt that it was the memory of J. W. Jenkinson’s and Geoffrey Smith’s teaching that caused Huxley to devote the whole of his laboratory research to experimental and analytical studies in ontogeny” (Witkowski 1992, p. 84). He looked at Huxley’s work on relative growth, while Ridley had looked at the study of embryology in Britain more broadly, with a focus on Oxford (Ridley 1986). But Baker, Witkowski and Ridley did not link Huxley’s Oxford education with his interest in the developmental action of genes.

What is novel here, then, is that I show that Huxley’s more expansive understanding of characters was nothing inevitable—it was rooted in his Oxford education. In a broader debate about the development of characters, Huxley’s tutors sided with what they called an “epigenetic” view, i.e., characters were end-products of context-dependent relative processes, rather than being pre-made in the material properties of the cell, such as its nucleus. Other theories debating the relationship between genetics, development and environment have been analyzed elsewhere (Sapp 1987; Müller-Wille and Rheinberger 2012). But Huxley’s own biological view about animal characters lacks such a history, which I show was rooted in his Oxford context. We will start with a brief exploration of Huxley’s discussion on the action of genes between 1942–1958, showing how Huxley distinguished characters from their complex interacting causes. Then I show that Huxley’s distinction was rooted in the preformationist versus. epigenetic debate that was pervasive during Huxley’s education at Oxford in the early 20th century. Through Huxley’s undergraduate essays, I show that he accepted the “epigenetic” idea. We will then see that Huxley read Weismann and others through that idea. I also demonstrate that Huxley made sense of socio-political issues in the 1910s no less through it. Finally, I show the effects of these early Oxford ideas in Huxley's later work in the 1920s and 30s. I demonstrate that Huxley debated against contemporaries, like the Neo-Lamarckian E. W. MacBride, and the geneticists William Bateson and Thomas Hunt Morgan, by employing his early “epigenetic” biology, implicit in his repeated argument that the expression “gene for” was only a shorthand, while characters were end-products of more complex “epigenetic” processes.

## Huxley’s View of Animal Characters, 1942–1958

Huxley made it abundantly clear that the alteration of gene expression was important for evolution. In the first chapter of *Evolution: The Modern Synthesis,* Huxley was careful to make a distinction between “variation” and “characters.” He wrote that variations were, broadly, of two types: “modifications” and “mutations” (Huxley 1942, p. 18). The former pertained to “alterations in the environment,” while the latter were “alterations in the substance of the hereditary constitution.” On the other hand, “characters” were a different business altogether. In all situations, those were combinations between heredity and environment, being products of both mutations and modifications. “Characters as such are not and cannot be inherited,” Huxley wrote, “[f]or a character is always the joint product of a particular genetic composition and a particular set of environmental circumstances” (Huxley 1942, p. 18). One could not, and should not, equate heredity with a character, as one should not equate the latter with the environment. To talk about a “genetic factor for a rose-comb” was to have a “one-to-one or billiard-ball view of genetics.” No character was “represented” by one genetic factor, or even a combination of factors. Huxley called this a “crude particulate view” that was “a mere restatement of the preformation theory of development: granted the rose-comb factor, the rose-comb character, nice and clear-cut, will always appear” (Huxley 1942, p. 19). We shall see that it is no accident that Huxley connected this “crude” view of characters with preformation.

In chapter three, he discussed, in a dedicated section, the relationship between genes and characters. He noted again that the notion of “Mendelian characters,” or of the inheritance of characters, should be dropped. There were multiple ways in which the expression of a gene varied. He noted pleiotropism, the view that a single gene may affect multiple characters, or the view, as I detail more below, that genes interacted with each other, which he owed partly to Fisher (Huxley 1942, p. 8). Another way was when genes interacted with the environment during the organism’s development. That is, “a given character represents the end-result of a great number of genes interacting with the environment during development, and is not inherited as such” (Huxley 1942, p. 63).

Although characters were products of broader relative processes, one gets a sense that Huxley put more emphasis on the role of genes and their interactions in the production of characters, in an asymmetrical sense. But this was no less true for Waddington (Loison 2022; Deichmann 2016). That did not hinder the latter from accepting a view of “reciprocal causation,” namely the view that phenotypes were jointly produced by genotype and environment (cf. Tabery 2014). One may distinguish here genetic *determinism* from genetic *reductionism:* while genes carried much weight in the making of characters, they were not solely responsible. Genes reacted in specific ways with the environment to produce characters (Loison 2022, p. 185).

Huxley’s commitment to a view of “reciprocal causation” can be seen in two similarities between his and Waddington’s work, evident in Huxley’s 1942 book. Just like Waddington, he emphasized the action of each gene as relative to other genes in a system, or a “gene-complex.” He wrote that:

[…] the most revolutionary change has come in regard to the way in which the expression of a gene can be altered by other genes. The discovery of this fact has given us the two fundamental concepts of genic balance and the gene-complex. Thus the internal or genetic environment of a gene may produce effects upon its expression which are as striking as those induced by the external environment, and of course very much more important from the point of view of evolution. (Huxley 1942, p. 64)

Indeed, natural selection did not act on any gene alone, but on a gene-complex. Doubtlessly this is why he focused chapter four on “Genetic Systems and Evolution.” Yet another similarity was Huxley and Waddington’s adoption of Thorpe’s “Organic Selection,” according to which the environment was not just a fixed entity selecting the organism, but the organism, in turn, consciously or unconsciously was choosing an environment, with evolutionary consequences (Radick 2017; Loison 2022).

Moreover, the two exchanged a friendly correspondence. Waddington, or “Wad,” as Huxley addressed him, was seemingly happy in 1957 about Huxley’s long-delayed knighthood. “I have always been shocked that one of the great liberal and progressive influences of our time has received little official recognition,” Waddington wrote to his friend. In turn, Huxley viewed Waddington’s *The Strategy of the Genes* favorably. The book has been hailed as an expression of Waddington’s criticisms against what he saw as the excesses of population genetics, including the passivity of the organism and a reductionism to genes (Loison 2022, p. 182; Peterson 2016). It should be telling that Huxley saw the book none the poorer. Indeed, Waddington was “glad you [Huxley] found something of interest in it and not too much that you strongly disagreed with.”^1^

Huxley openly adopted Waddington’s “epigenetics” in his lesser-known book *Biological Aspects of Cancer* of 1958. There he argued that, “there is no single ‘cause of cancer’’’ and hence one should expect a rich taxonomy of different cancers. He wrote that one cause of cancers was “epigenetic” which was “the analytic study of individual development” (Huxley 1958, p. 94). Citing Waddington’s 1956 *Principles of Embryology*, he said that Waddington tackled the “central problem of differentiation” and showed that much more was needed to understand the “method by which tissues and organs differentiate in the course of normal development” (Huxley 1958, p. 94). When he discussed the genetics of tumors, he repeated the point he had made at the start of his 1942 book, namely that “characters as such are never transmitted, but that their manifestation depends on the interaction of a large number of genes with each other and with the external and internal environment” (Huxley 1958, p. 36). Huxley’s adoption of Waddington’s “epigenetics” only in 1958 should warn us against equating the latter’s term with what Huxley and his tutors called, as I show below, the “epigenetic” idea (see Deichmann 2016). Huxley’s “epigenetic” view should be understood in the context of a debate emerging at Oxford with the “modern” preformationist ideas in the early 20^th^ century.

## Oxford Biology and the “Epigeneticˮ View, 1899–1906

Walter Frank Raphael Weldon is usually thought of as a biometrician, but he was also an embryologist when he became the Linacre Professor of Zoology and Comparative Anatomy at Oxford between 1899 to 1906 (Provine 1971; Radick 2023). When Weldon arrived at Oxford in 1899, Poulton, a fellow Oxford colleague, had already formulated his support for Weismann’s theory of the continuity of “germ-plasm,” the hereditary material passed unchanged from generation to generation (Poulton 1908, p. 127). But Weldon, more so than Poulton, was careful not to reduce the entire process of character development to the germplasm. Why? Before 1899, Weldon had studied under F. M. Balfour, and then worked as an embryologist (Radick 2023). At that time, Ernst Haeckel’s view that phylogeny caused much of ontogeny, that ontogeny recapitulated phylogeny, was influential (Haeckel’s brand of this theory was his famous “biogenetic” law). To Gould, Balfour was for the most part a follower of Haeckel’s view (Gould 1977). Churchill has shown, however, that Balfour eventually realized that ontogeny could itself be creative of evolutionary novelties, as during development variations and adaptations could arise (Churchill 2007).^2^

In 1882, Balfour’s pupil, Weldon, noted non-recapitulative variations in the development of the lizard *Lacerta muralis* (Weldon 1883). But, by the 1880s and 1890s, advances in cytology meant that development was increasingly understood through the cell’s mechanics (Maienschein 2007; Müller-Wille and Rheinberger 2012). Wilhelm Roux and August Weismann influentially argued that the development of characters mostly followed the material properties contained in the cell’s nucleus.^3^ Differences of cell-development were understood through the differences of those material properties, said to emerge as development occurred (Maienschein 1991). If the cell’s internal material properties divided according to a developmental sequence determined by phylogeny, ontogeny *de facto* recapitulated phylogeny through the sequential division of cellular properties. The biogenetic law thus seemed to resurface.^4^ The embryologist Hans Driesch famously rejected the view that differences in cell development could be explained solely via differences of the cell’s material properties, claiming that the cell’s material properties remained the same throughout development. Differences of cell-development should be understood through the reactions of the cell’s material properties to its external conditions, that differed from cell to cell (Maienschein 1991).

Nevertheless, by the early 1900s, William Bateson appeared to revive the debate by seemingly claiming that observable characters paralleled factors in the nucleus. Weldon saw Roux, Weismann and Bateson to be sailing the same boat. In an unpublished manuscript mainly written in 1904–1905, Weldon rejected what he called the “determinants” of Roux and Weismann, laying the groundwork for his criticism against Bateson.^5^ He favored Driesch’s view that cells differentiated relative to the environments acting on them. More so than Driesch, Weldon also noted the importance of the internal environment of the cell. The observed and eventual “dominance” – end-form – of any cell was partly due to the developmental possibilities accumulated from the past in the nucleus interacting with different environments (cf. Radick 2005, pp. 35–6). Some developmental possibilities were restricted “due to a direct inhibition of certain properties, through the influence of neighbouring cells, rather than to any change in their material constitution.ˮ^6^ Weldon’s pupil, Jenkinson, less inhibited to put labels than his teacher, called what he was against “preformation,” and what he favored “epigenetic evolution.”

There is no evidence that Jenkinson read Weldon’s manuscript, but I show that he supported ideas similar to Weldon’s. Jenkinson has received some attention in the literature, but not much in relation to Weldon’s work (Gould 1977; Ridley 1986; Sapp 1987; Maienschein 1991; Horder 2008). With a degree in the humanities (*Literae Humaniores*) from Oxford in the early 1890s, he went to study with Weldon at University College, London, after 1894 (Marett 1917). In 1900, in a paper on the development of the mouse, he attacked the doctrine of recapitulation. Based on his observations of the mouse’s development, he noted the influence of cytoplasmic “organ-forming stuffs” on the nucleus, concluding that “I believe that all attempts to institute homologies […] are foredoomed to failure” (Jenkinson 1900, p. 75).^7^ The development of mammals was *sui generis*. i.e., unique in every case. Jenkinson had acknowledged that “Professor Weldon’s laboratory at University College, London” was crucial for his work (Jenkinson 1900, p. 77).

Between 1901 and 1905, Jenkinson finished his doctoral work at Oxford and, in 1906, he published a series of papers emerging from it. He now focused more on the cell theory, specifically with regards to Roux and Weismann. At the end of a paper on the “Germinal Layers of the Vertebrates,” he placed Roux and Weismann’s theories in a wider context. He contended that “the Roux-Weismann hypothesis of preformation” is a “modern resuscitation of the famous theory of evolution which was destroyed by Wolff more than a hundred years ago” (Jenkinson 1906, p. 82). Jenkinson argued that preformation was compatible with recapitulation. If the cell was preformed by its nucleus, the “prime cause of differentiation – the structure of the fertilized ovum – is itself a heritage from a long line of ancestors, [and] each individual will of necessity repeat in its ontogeny the history of its descent” (Jenkinson 1906, pp. 82-83).

He rejected both preformation and recapitulation. He argued that, if one wished to retain the word “recapitulation,” one should not use it as a “recapitulation of any adult ancestral type,” i.e. in Haeckel’s sense. It should be used in the sense of cells retaining the developmental possibilities of their ancestors, as “merely a repetition of similar ontogenetic functions by cells which have inherited a similar structure. In destiny, however, such cells may be exceedingly diverse” (Jenkinson 1906, p. 87). Jenkinson argued that there were two problems that the “experimental embryologistˮ had to face. Firstly, to “describe in accurate terms the influence exerted upon the embryo by its environment” and the second, “to determine the mutual relations which subsist between the parts of the embryo and between the parts of the whole” (Jenkinson 1906, p. 88-9). That is, “what are the external and what the internal factors which govern the process of differentiation” (Jenkinson 1906, p. 89). “Physiological study,” Jenkinson wrote, was important to understand development, which might allow the experimental embryologist to better grasp “the problem of the epigenetic evolution of the complexity of the adult form from the apparent simplicity of the fertilized ovum” (Jenkinson 1906, p. 89). The idea of “epigenetic evolution,” that internal and external factors interact to evolutionarily shape development and characters, was no less adopted by another of Weldon’s pupils, namely Geoffrey Watkins Smith.

Born in 1881, Smith studied with Weldon at Oxford, gaining a degree in Natural Sciences in 1903, before going to Naples until 1905, when he started working as demonstrator and lecturer in the Oxford University Museum (Anon 1916). His first study was on the *Rhizocephala*, parasitic castrators abundant in Naples, whose usual hosts were crabs, the latter also attracting Weldon’s attention. When *sacculina*, a species of the *Rhizocephala*, infected the crab, the crab hosts’ gonads degenerated, or barely developed. While female crabs still developed (if modified) secondary female characteristics, Smith observed, males developed only degrees of female characteristics. The male either ended up with a mixture between male and female characters or with complete female sexual characters, depending on the time when the *Rhizocephala* infected the male crab (Smith 1906, p. 82).

Smith concluded that the development of secondary sexual characters could not be determined by the gonads, because the secondary female characters in the male crab developed without an ovary (Smith 1906, p. 84). He contended that an inherited “potentiality” of forming female sexual characters remained in the male, and it might become active under specific conditions, such as when a “sexually formative substance” might act in specific ways. The gonads, then, did not preform sexual characters, but characters were instead products of specific interactions between inheritance, the gonads, and body substances. The eugenical implications of this developmental flexibility have already been analyzed in depth (Brooks 2021, 2023).

What is important is to consider how Smith conceived the biological development of sexual characters. He noted that the formative substance might have different effects on cells depending on the time of the interaction between the substance and the cells. Why did only some cells differentiate into ova or sperm upon contact with this substance? He reasoned that only those cells “in a particular position and of a particular internal structure are capable of becoming ova under the influence of the sexual formative substance” (Smith 1906, p. 85-6). In other words, the development of sexual characters was a result of an interaction between the sexual formative substance and the state of the cells at a particular time. Smith wrote that his theory was a “hypothesis” that was compatible with “modern experimental embryology,” a “sympathetic grasp,” as he called it, “of both epigenetic and evolutionary ideas in embryological theory” (Smith 1906, p. 86). He fell well in line with Weldon and Jenkinson’s ideas. The stage was thus set for Huxley’s arrival at Oxford.

## Huxley’s Reception of “Epigeneticˮ Biology in His Undergraduate Years, 1906–1907

Huxley adopted his tutors’ “epigenetic” ideas in his undergraduate work. He arrived at Oxford in autumn 1906, after Weldon had just died in April 1906. His personal tutor at Oxford was Smith, and Jenkinson was his lecturer in experimental embryology (Witkowski 1992). No evidence has been found from 1906-1907 that shows the nature of Huxley’s interactions with Smith. The earliest known notebook with Huxley’s notes on Jenkinson’s lectures, where he wrote some remarks, for instance, on *Lacerta muralis*, was written in 1907 or 1908.^8^ A series of essays Huxley drafted in 1907, however, suggest that Huxley used Jenkinson or Smith’s epigenetic ideas by 1907.

After finishing his studies at Eton and gaining a scholarship at Balliol, Oxford, Huxley met Driesch in the summer of 1906, who by this time had turned to his version of vitalism (Huxley 1970, p. 61). Coming to Oxford in autumn 1906, he had also met the physiologist and philosopher John Scott Haldane around the autumn of 1906, who had been criticizing vitalism (Huxley 1970, p. 65). Jenkinson had, in turn, acknowledged in 1906 the help of “Dr. Haldane” (Jenkinson 1906, p. 1). Haldane, who often attended the so-called Oxford Biological Club, argued both against Driesch’s vitalism and against reductive materialistic theory. He was proposing a version of the “organicist” philosophy which aimed to show that characters were the product of the organism as a whole, rather than its parts (Sturdy 1987; Esposito 2013; Peterson 2016). Haldane would later figure in one of Huxley’s essays.

But Huxley paid equal attention to the mechanistic theories of his contemporaries. Huxley read Jacques Loeb’s *Comparative Physiology of the Brain and Comparative Psychology* (1900), which showed that the brain, although complex and seemingly “vitalistic,” was “determined” materially and could be explained, as Loeb put it, mechanistically, i.e., without recourse to a “mystery” (Allen 1975).^9^ As we will see, however, Huxley also saw evolution and the whole organism to be crucial. Natural selection preserved complex “epigenetic” interactions within an organism, being his ticket to understanding intricate organisms naturalistically, simultaneously seeing them more than simple physico-chemical machines and less than a product of vitalistic forces.

Writing an essay on natural selection, Huxley explored the different theories about the “origins of variation,” which he categorized as either gradual or sudden.^10^ As an example of sudden variations, he mentioned Hugo de Vries’s mutation theory, while of the gradual he gave the example of “Weldon’s work with crabs.” Although he thought that both were important, he leaned towards gradual variation, since small variations allowed for the maintenance of a “harmony” between parts, while large variations disrupted harmonious organisms. In another essay, on the development and functions of the brain, he used more explicitly his tutors’ epigenetic ideas.

To understand the brain, one had to understand the development of its cells. Huxley held that the fate of any cell was not preformed by its material properties, or a vitalistic mystery, but could be explained in terms of living “epigenetic” processes. Citing Loeb’s 1900 book, he began his essay with what was virtually a ladder of complexity of the nervous system, from the “Geotropism of Root,” with “v. little specialization,” to more complex organisms.^11^ The point was that in different organisms cells came in different relations with other cells and the organism. But Huxley went on to discuss the development of the brain in general, and the development of cells, emphasizing how interactions between the cell’s internal and external environments could explain its development.

Cells specialized and divided in labor depending on their “arrangements,” i.e., on the collections of cells and their relations. He listed the different specializations which cells could adopt, such as becoming “fibres” and being able to “conduct external stimuli from a sense organ throughout the body,ˮ allowing some “Protozoa …to perform coordinated movements as a whole in response to a localized stimulus.”^12^ In all cases, the actions of “ordinary protoplasm” were not “determined” by the protoplasm’s properties but “by the arrangement of parts.” Huxley wrote that a specific specialization did not mean that a cell had completely lost “the other functions possessed by the “primitive cell” (which he also called “undifferentiated protoplasm”). He said that, since the “relative processes” and “relative positions” were important for the destiny of any cell, “the result is mainly given potentially in the germ & partly determined by varying outer influences.” He defined the way cells specialized as an “epigenetic idea:” the action of any part was dependent on its location, and its relations to its internal and external parts.^13^

The fate of the cell’s internal properties could be changed by any generational alterations, be they hereditary, developmental or environmental. Evolution could thus happen in many ways. According to Huxley, “each generation is mainly determined by the generation before, but if it should itself alter in certain ways, these alterations will cause alteration in the next & all subsequent generations.”^14^ That being so, no specific process determined evolution, but any change in one generation could have significant consequences upon the next. Huxley believed that “the development of the race is therefore like that of the individual, epigenetic.” “What the zoologist calls epigenetic processesˮ was therefore characteristic of both development and of evolution (original emphasis).^15^ It was in this scheme that one should see the place of “natural selection.” The cell’s evolutionary material properties yielded different developmental potentialities relative to various environments. Natural selection preserved the internal material properties which could yield good developmental potentialities in specific environments, so that the “organism becomes a storehouse of purposeful arrangements.”^16^

Huxley wrote that such an account dispensed with any “reference to or consciousness of any ideal purpose or goal,” or even a “higher grade of mystery.”^17^ For example, one could thus understand the complex vertebrate “system.” Regarding the vertebrates, Huxley noted down on the margins of his essay “qu. JSH,” J. S. Haldane. The latter aimed to show that complex self-regulating organisms were neither preformed nor products of a vitalistic force. Their parts were integrated in “co-ordinated wholes” whose operations, although naturalistic, were distinct from pure chemistry and physics (Sturdy 1987). Huxley had a similar ambition. A selection of “epigenetic” arrangements, between parts within and outside the organism having a chain-reaction upon cells’ internal material properties, could not be equated with simple physico-chemical mechanisms, as internal and external interactions did not just obey physical and chemical laws. Neither could they be equated with a vitalistic force, no matter how purposeful an organism might seem. Dynamic interactions between internal and external units at different levels explained development and evolution. Huxley understood Weismann no less through this framework.

## Huxley’s Reception of Weismann, and Jenkinson’s Experimental Embryology, 1908–1912

We know from Peter Bowler that there was a continuous “faith in Darwinism” at Oxford against Lamarckian ideas, not least represented by Poulton (Bowler 1983, p. 30; Meulendijks 2021). Poulton became an adept of Weismann’s ideas, and, by 1908, Huxley read Poulton’s 1908*Essays on Evolution*, and then Weismann. We can find diary notes in a notebook where, on the 8th of November 1908, Huxley wrote that “after breakfast read a lot of Weismann – finished the first vol. [of *The Evolution Theory*] – it is almost all very convincing.ˮ^18^ We can see what particularly struck Huxley in an essay written just after, titled “Natural Selection & Lamarckism.ˮ^19^ Darwin’s much-maligned theory of “pangenesis,” Huxley remarked, as all Lamarckist theories, upheld the view that external conditions’ “act directly on the germ-plasm.” This exposition closely resembled Poulton’s essay “Theories of Heredity.” Like Poulton, who was citing his colleague Jenkinson, Huxley disagreed with the direct modification of the germ-plasm, as if by the conscious will of organisms, but argued that use and environment could change how the germ-plasm reacted to dynamic situations.

Different “determinants” (Huxley put the word between inverted commas) in the germplasm could be strengthened or weakened by “use,” and the more they were strengthened, the more “nutriment” they received. This explained to Huxley how, without Lamarckism, traits could degenerate or could become oversized. The non-germplasm-modifying “indirect action of externals,” e.g., nutriment during development, Huxley concluded, “is probably considerable,” especially given recent research in “organ-forming stuffs circulating in the ovum, & by the assumption of similar stuffs necessitated by the effects of parasitic castration.ˮ^20^ Jenkinson and Smith’s works reverberated thus no less in Huxley’s reception of Weismann. The lesson remained that, in between heredity and the final character, there was an “epigenetic” process of development and physiology which could change the character, with significant evolutionary effects. With less use, a character could degenerate. When this happened, different inherited possibilities could be brought out, a lesson that Huxley had learnt from Jenkinson and Smith. That is not to say that Huxley completely agreed with his tutors. Jenkinson’s emphasis on the role of cytoplasm, for instance, which has been shown by Jan Sapp, was barely discussed by Huxley (Sapp 1987). But the broad epigenetic ideas were taken up.

Note that Huxley adopted Weismann’s notion of “germinal selection,” according to which there was a struggle in the germplasm between “determinants,” a variant thereof also adopted by Roux (Swiatczak 2023).^21^ With a different somatic environment and different use of organs and traits, a determinant might increase its expression through more nutrition, and thus the struggle between determinants within the germplasm would yield different results. Given that the struggle was context dependent, Huxley did not think a character could be understood as the sole result of either heredity or environment alone, being neither just inherited, nor just acquired. Although, as explained previously, Weldon died before Huxley arrived at Oxford, Radick has shown that the idea that characters were both inherited and acquired was “something of a motto for Weldon” (Radick 2024, p. 109).

None of this, regardless, could have prepared Huxley for the events of November 1908, when his mother died of cancer. Only after several months, on March 15th 1909, did he finally bring himself to face it. “Dearest mother,” he began, “I can at last write my memory of you, in the hopes that I may cleanse myself – I know what you have loved, what you would have hated, but I can bring it more home, can keep it, more real & effective by putting it down in black-&-white. Now let me tell you [that] I am going to do it every day.”^22^ Through this type of epistolary therapy, we get a glimpse of Huxley in 1909, in his final months at Oxford. On April 1st, 1909, he wrote that he had a “quite good day – I did a reasonable lot of work, kept at it by Jenkinson’s extraordinary keenness & energy, & it was a lovely day.” In the same month, Huxley received a copy of Jenkinson’s *Experimental Embryology* of 1909*.* He read it through, richly annotating it.

Jenkinson’s framed his book as part of a long-running debate, from Aristotle to Weismann, of “Epigenesis or Preformation.”^23^ Huxley underlined Caspar Friedrich Wolff’s *Theoria Generationis,* that Jenkinson said “arousedˮ evolutionists from their “dogmatic slumbers,” namely Charles Bonnet’s preformation (Jenkinson 1909, p. 14). The “modern” versions of Bonnet’s preformation theory, Jenkinson wrote, were, again, Roux and Weismann’s theories, whose names Huxley also underlined (Jenkinson 1909, p. 16). The alternatives, to be expected, were Driesch and Curt Alfred Herbst, whose names Huxley equally underlined. “Once again,” Jenkinson wrote ending his introduction, “we find ourselves face to face with the old alternative, Preformation or Epigenesis; and it is to the desire of solving this problem that a very considerable proportion of modern experimental research is attributable” (Jenkinson 1909, p. 19). Jenkinson was clear that epigenesis was “the fundamental expression of developmental fact” (Jenkinson 1909, p. 16). It was the basis, but it risked being too descriptive. It needed to be supplemented by “modern experimental embryologyˮ which investigated “cause and effect” (Jenkinson 1909, p. 17).

One remark that Jenkinson made was that one should distinguish between a “predetermined” embryological process and “preformation”, which was the crux of the whole matter about how one should conceive of change in development (Jenkinson 1909, p. 20). He wrote that it was obvious that there was “in some sense a predetermined process” in development, namely that development partly depended on the internal material properties of the cell, which were there before development had even started (Jenkinson 1909, p. 20). This “causal explanation” did not presuppose “the preformation in the germ of morphological units representing every possible inheritable character” (Jenkinson 1909, p. 20). This predetermination-without-preformation emerged, Jenkinson wrote, “in Herbst’s and Driesch’s conception of the events of ontogeny as so many responses to stimuli exerted by the developing parts on one another” (Jenkinson 1909, p. 20). One particular thing about possible stimuli for the cell’s material properties that Huxley noted from Jenkinson’s book was the role of factors in the cell’s cytoplasm, the “organ-forming substances,” from Edwin Conklin’s 1905 research.^24^ Jenkinson’s distinction between predetermination and preformation, with its accompanying emphasis on experimental epigenesis, reverberated in Huxley’s subsequent work.

## The Continuation of Huxley’s “Epigeneticˮ View in His Wider Work, 1912–1916

It is worth stressing that Jenkinson’s experimental version of epigenesis was not the same as other versions of epigenetic views of development. For instance, taking cues from Karl Ernst von Baer, Thomas Henry Huxley’s epigenetic view supposed that the gradual development of the organism from undifferentiated matter occurred through “the action of molecular forces” (Richmond 2000, p. 277). Therefore, although one might say that a child, a teenager, and an adult are different individuals, to T. H. Huxley they were one individual having different forms, produced by the same molecular forces that arose at the start of development. Those forces came from “a single egg taken togetherˮ (Foster and Lankester 1898, p. 150). But to his grandson, Julian Huxley, epigenetic development also entailed the interaction of internal parts with the environment.^25^ These dynamic interactions produced changes that could arise at any time during development, making different individualities such as when a tadpole transformed into a frog. In his 1912 *The Individual in the Animal Kingdom*, Huxley equated his grandfather’s view with the “mutations” of Hugo De Vries, because he saw that changes to development could only realistically arise during “sexual fusion” (Huxley 1912, p. 80). He wrote that “it is found that not all mutations are similar to those we have described: permanent and considerable changes may take place at any time during the life-cycle, and not in the sexual act aloneˮ (Huxley 1912, p. 81). Like Smith and Jenkinson, Huxley adopted a view of “epigenetic” development that dispensed with forces and that saw the gradual construction of the organism as modifiable at any point in the life-cycle.

Huxley’s epigenetic biology, also emerged in his understanding of co-operative evolution. In his *Individual in the Animal Kingdom* (1912)*,* Huxley noted how natural selection could act on single individuals, as it could act on individuals dividing in labor and co-operating to survive. Suppose, he wrote, that, instead of a struggle between two organisms to the death, in the “Economy of Nature, the two should conspire together to create a vacuum of their own” (Huxley 1912, 134). For, “though it is undoubted that the pressure of the struggle is always forcing life into these vacuums of vacant spaces, we have to look further before we find what the effect on life will be” (Huxley 1912, p. 134).^26^ Competition between individuals was a way the struggle occurred. Another way was cooperation through “division of labour,” meaning that single individuals specialized differently relative to each other, working together to survive the struggle for existence. Evolution was thus not just a competition between different heredities, but organisms could boost their characters via new relations and activities with other animals.

This view played a pivotal role in Huxley’s 1916 Extension Lectures at Rice University, where he worked between 1913 and 1916. Asked to deliver a set of lectures open to the public, Huxley chose to apply biological knowledge to the issues of the day. As the Great War was raging, Huxley formulated, in his lecture “Biology and War,” a biological argument against war, arguing that human characters, including behaviors, were products of an epigenetic evolution. It is true that Huxley saw the war as dysgenic, no doubt partly lamenting the death of both Jenkinson and Smith between 1915 and 1916. War was, however, “dysgenicˮ not just because people of talent died, but also because nationalism impeded the making of more advanced human organizations that could push the specialization and differentiation of human parts further. Huxley wrote that “co-operation and community brought out in the ancestor of man all those essentially human & higher qualities which would never have appeared had he remained content with solitary existence.”^27^ For the same reason, Huxley wrote that women should be given equality of opportunity, since mutual selection between the sexes would increase both their qualities (Bartley 1995). Biologizing social diversification, he even supported democracy on biological grounds (Sommer 2014).

It was nevertheless to be a centralized democracy. Only by adding an international “organˮ could evolution proceed. Huxley quoted President Taft’s “League to Enforce Peace.” He wrote that, at some point, a future scheme “shall be truly international, carried out by co-operation between states, not by one state enforcing its will on others. In other words ... the nation will no longer be the greatest unit of the human race.ˮ^28^ The need for an organ that enforced international cooperation underscored Huxley’s epigenetic view of the evolution of human societies. For nations had arisen through processes of increasing division of labor and coordination, just as they could evolve through the future extensions of such processes by coordinating through a central organ. “Line upon line, precept upon precept,” referring to the evolution of social organizations, “is the rule of organic development: biologists call it epigenesis.”^29^ Epigenetic evolution was the basis for human societies and their advancement. Based on a view of biological epigenesis, Huxley therefore argued for a vision of evolution based upon diversity and cooperation leading to greater specialization and democratization (Sommer 2014).

## Huxley’s Epigenetic Biology Against MacBride’s Lamarckism and Bateson’s Mendelian Genetics, 1921–1925

Huxley’s epigenetic biology also had repercussions on his work in the 1920s. At that time, the embryologist E. W. MacBride’s defended the Lamarckian thesis of the inheritance of acquired characters by arguing that genetics entailed a discredited preformationist view where Mendelian factors determined characters. In an article aimed against MacBride’s pro-Lamarckian claims that genetics entailed age-old preformationism, Huxley defended the epigenetic view that genes only produced characters when reacting with the environment. Echoing Driesch, Weldon, and Jenkinson, he wrote that “it should not surprise us in the least to find the identical gene-mechanism in every cell of the body.” The “gene-mechanism” worked differently in different environments. Piano mechanisms might be the same, but the melodies will differ depending on the pianist or “executant” (Huxley 1921, p. 246). The “gene-mechanism,” or “gene-complex” was the piano mechanism, while the “executant […] is represented by the environment […] whether intra-cellular, intra-organismal, or external” (Huxley 1921, p. 246). Every cell, indeed, was a “system.” He put forward the view that “each cell represents a particular state of equilibrium, and the organism as a whole an equilibrium of all the cells with each other and with the environment. [...] The development of an organism is a series of states of equilibrium, usually of increasing complexity.” Huxley argued that “from this standpoint the unity of the organism as a whole is not only explicable, but necessary” (Huxley 1921, p. 247).

The merit of Weismann, he argued, had been to dispute the inheritance of acquired characters. But his demerit was “his total failure to construct a physiological theory of development” (Huxley 1921, p. 247). Just two pages before, where he began discussing, as he called it, the “translation of the [genetic] constitution, in certain conditions of environment, into the adult organism,” he cited Jenkinson’s 1909 book (Huxley 1921, p. 245). In the same vein, Huxley supported Richard Goldschmidt’s “physiology of sex determination,” arguing that “the earlier rigid belief that sex-determination was entirely a matter of the chromosome-constitution must” be modified (Huxley 1923, p. 928; Richmond 2009).

In 1924, Huxley co-authored an article with the biologist and sociologist Alexander Carr-Saunders attacking MacBride, calling his Lamarckism a form of “preformation.” Carr-Saunders had studied zoology at Oxford from 1905 to 1908 but then chose to pursue sociological problems (Blacker 1967, p. 365). In 1922, he had published *The Population Problem: A Study in Human Evolution*, where, in a chapter on “Environment among Animals and Plants,” he cited Jenkinson’s work claiming that heredity and environment were “complementary” to each other (Carr-Saunders 1922, p. 326). He quoted Jenkinson asking his readers to think that, if they were to remove one environmental element from development, “the specific typical end which is reached in normal development will not be attainedˮ (Carr-Saunders 1922, p. 326). In their co-authored article, Huxley and Carr-Saunders called MacBride and his allies’ inheritance of acquired characters “preformationˮ because the germ-plasm modified by the environment seemed to produce characters in offspring ready-made, without complex interactions with other parts. Huxley and Carr-Saunders’ alternative was epigenetic. They wrote: “It looks much more as if a chain of reactions was in each case [of development] set going which moved toward an appointed end, but an end not necessarily resembling any of the substances present at the beginning” (Huxley & Carr-Saunders 1924, p. 231). In the same vein, Huxley criticized T. H. Morgan’s 1926 *The Theory of the Gene* in a review he tellingly titled “A Static Theory of Heredity.” He noted that Morgan had not made it clear enough that a character “emerges as the clear-sighted have always seen it - a resultant of a large number of inner agencies, inter-acting with a large number of outer agencies. The term ‘unit character’ should never have been used” (Huxley 1926, p. 581).

It was around this time that Huxley exchanged correspondence with William Bateson (1861–1926). Triggered in the context of Huxley co-founding the *British Journal for Experimental Biology*, the dispute between Huxley and Bateson underscores the former's support for all-round “epigenetic” biology (Erlingsson 2013). On June 25, 1925, Huxley, in response to a meeting of the *Genetical Society* that had just taken place, Huxley wrote to Bateson that he was excited about how the meeting had gone. But he had his doubts about the methods people there employed, specifically the “factorial method.” “It seemed to me,” Huxley wrote, “that in many of your problems you were getting to a point at which what I might call the factorial method would give out, or at any rate yield rapidly diminishing returns.”^30^ He proposed that the “physiological method might yield enormous advances.” Huxley specifically disagreed with some of the people at the meeting discussing “genetic entities” as if they actually encapsulated traits like “maleness” and “femaleness.”

Responding to Bateson in December 1925, Huxley gave the example of the aquatic plant *Ranunculus aquatilis* to emphasize his idea of how characters came about, and also how genes could contribute to them. Take “dd” to mean the genetic factors “for the development of dissected leaves” in the plant. Huxley argued that “dd in the presence of water habitat ->dissected leaves.” While dd in the presence of a land habitat “->entire leaves.” “Here,” Huxley continued, “the external environment determines the expression.” If the interaction with the external environment was very important in the case of these aquatic plants, the “internal genetic environment,” he wrote, “can be equally important.” Huxley generalized his point about interactions, saying that “what genetics needs is what physiology has woken up to in the last quarter-century, the fact that ...the action of a factor is a meaningless abstraction – for its expression depends on interacting with the rest of the genetic constitution. Factors themselves may be, and doubtless are, definite units, but their expression involves ‘the organism as a whole’” (original emphasis).^31^ Showing just that, Huxley worked with his pupil E. B. Ford on the amphipod *Gammarus chevreuxi.*

## Genetics, Development, and the “Gene For” Shorthand: Huxley’s Work Between 1925 and 1936

Huxley and E. B. Ford’s work on *Gammarus* has received some attention. Stephen J. Gould saw the work on *Gammarus* as a turning-point from 19th to 20th century biology (Gould 1977). Peter Medawar has hailed it as a prime example of the impossibility to distinguish nature and nurture in the determination of IQ (Medawar 1982). Robert Olby has noted Huxley’s interest in developmental gene actions in *Gammarus*, but did not link this back to his Oxford context (Olby 1992). The eyes of *Gammarus* could develop various shades of color, which previous investigators had attributed to genes. But Huxley and Ford found that they could modify color-development by changing the temperature. However, in a note in *Nature* in 1925, Huxley and Ford wrote that “very little is known as to the developmental mechanism by which this correlation [between genes and characters] is brought about. We thus have accurate pictures of the gene-complex and of the character-complex, but these pictures are, we may say, static, and the dynamic relations between the two are obscureˮ (Huxley and Ford 1925, p. 861). On March the 31st 1928, Huxley reported to Frank R. Lillie that “we are getting some nice stuff with Gammarus” which is “helping to bridge the gap between genetics and development.”^32^ Ford remained sensitive to the distinction between genes and characters, later writing, in a Jenkinsonian way, that “all characters are the combined results of genes acting in a given environment. Alter the genes or the environment and variation may ensue” (Ford 1931, p. 30).

In response to MacBride, Bateson, Morgan, and their supporters, Huxley began reiterating that the expression “gene for” was a shorthand for a more integrated process of biological character-making. In the *Science of Life*, written with H. G. Wells and G. P. Wells, Huxley lamented that “we grow accustomed to using convenient but in a sense misleading shorthand phrases like ‘the gene for blue eyes,’ or the ‘albino gene’, that we tend to think of the genes as in *some way little replicas of the characters* with which they are concerned, and of the gene-outfit as being a sort of compressed organism, with a point corresponding to each part of the body” (Wells et al. 1931, p. 324; original emphasis). But this idea was “wholly false.” It was “really a survival of the preformationist ideas of the eighteenth century, which so worked on the imagination of one microscopist that he actually drew a human sperm with a *homunculus*, a miniature man, squatting within the head!” (Wells et al. 1931, p. 324, original emphasis).

By 1934, when Huxley co-wrote his *Elements of Experimental Embryology* with De Beer, the point had already become old. They argued that “the modern view, which combines an epigenetic outlook on development with the particulate theories of neo-Mendelism, denies any such simple correspondence between hereditary germinal unit and developed adult character.ˮ To understand characters, one had to understand how they developed relative to their functions. And “the *function*” they wrote, was analyzed by the “rather special branch of embryology usually called physiological genetics” (Huxley & De Beer 1934, pp. 4–5, original emphasis). Huxley carried this rather expansive view of the making of characters onto his 1936 Galton Lecture. It is true that to Huxley it was important to equalize the environment so as to “encourage favorable mutations” to emerge. But this presupposed that the environment also had an active role in shaping what characters were developed. In a section of his Galton Lecture subtitled “Environment and the Expression of Genetic Traits,ˮ Huxley argued against a preformationist view of genetics. “Characters are not and cannot be inherited,ˮ he wrote, “in the sense in which inheritance is used by the geneticist. What are inherited are genes, factors, genetic outfit. Any character whatsoever can only be a resultant between genes and environment.” Again, “a gene for white flower-colourˮ was only “a shorthand notationˮ (Huxley 1936a, p. 14). Therefore, doing proper eugenics also meant good social epigenesis. Only a “social systemˮ that encouraged “social traits such as altruism, readiness to co-operate, sensitiveness, sympathetic enthusiasm and so forth,ˮ and which provided “nichesˮ for different specialization, could allow for a better expression of genes (Huxley 1936a, p. 28). Relative interactions between heredity, other people, their development/education, and the socio-political environment, brought about characters. While it is true that Huxley targeted this against growing movements in Germany and was in this sense a “reform eugenicist,” his underlying biological views stretched back all the way to Oxford (Allen 1992).

The same point, that characters are end-products of interacting processes, was repeated by Huxley in 1936 in his well-known “Natural Selection and Evolutionary Progress.” There he wrote, again, that Mendelian *characters* do not exist, because no gene had constant effects. Drawing on Fisher’s work, he argued that a gene can become “dominantˮ through other modifier-genes. But this was part of Huxley’s wider argument that the development of characters was a product of complex interacting epigenetic processes. Developmental processes could change the effects of genes just as much as other genes did. He argued that “a large number (possibly the majority) of genes exert their effects through the intermediation of a process operating at a definite rate,ˮ a rate which was “relative – relative to the speeds of other processes of development and development in general” (Huxley 1936b, p. 92). Development was not forgotten, nor was gene-development reciprocal causation. This brings us back to 1942, when, again, Huxley wrote that “a study of the effects of genes during development is as essential for an understanding of evolution as are the study of mutation and that of selection” (Huxley 1942, p. 8).

## Conclusion

With his biology rooted in his Oxford education, Huxley viewed the making of animal characters as part of a wider “epigenetic” biology that encompassed dynamic relations between internal and external factors. Rather than a “Mendelian Revolution” in Huxley’s thinking, there was a continuation from development to genetics (see Hodge 1990). Emphasizing the natural selection of genes, and gene systems in his 1942 book was, this paper shows, just that: a matter of emphasis. We have clues as to why Huxley wrote the book in that way. It has been argued that his endorsement of Fisher’s gene-focused mathematical equations gave him a way to unify the biological sciences in a coherent evolutionary narrative that embraced his wider views about “evolutionary humanism” and progress (Smocovitis 1996, p. 139). Heeding calls to further historically contextualize Huxley’s broader synthesis, here I have focused on another aspect of Huxley’s thinking: the Oxford origins of his early 20th century epigenetic biology. We would benefit from a fuller treatment of Huxley’s biological thinking no less, for, as Cain has noted, Huxley’s various ideas should be considered together rather in isolation, as “no one synthesis stood out for him as more important than the others” (Cain 2010, p. 372).

## Data Availability

No datasets were generated or analyzed during the current study.

## Footnotes

1 C. H. Waddington to Huxley, January 1, 1958. Box 26, Folder 1, “General Correspondence.” Julian Sorrell Huxley Papers (1899-1980). Woodson Research Center, Fondren Library, Rice University, Houston, Texas. Hereafter abbreviated to Huxley Papers.

2 For more on Balfour, see Geison (1978).

3 Churchill reminds us, however, that Weismann’s emphasis on the nucleus did not mean that he reduced the development of the organism to it (Churchill 2015). Later identification of Weismann as a “preformationist” thus suggests we should separate Weismann from Weismann*ism*,

4 See Churchill (2015) for Weismann’s friendship with Haeckel and his complex adoption of Haeckel’s ideas. See especially the discussions on pp. 54-67 and pp. 104-107.

5 For more on Weldon against Bateson, see Radick (2023) and Shan (2020). Walter F. R. Weldon, 1904-5, “Theory of Inheritance*.* II. Experimental Results Tending to Support the Galtonian Conception of Conditional Dominance.ˮ Chapter Four, “Dominance and mutilation in embryo and larvae.ˮ Unpublished MS, Pearson/5/2/10/4, Karl Pearson Papers, University College London Archives, London.

6 Walter F. R. Weldon, 1904-5. “Theory of Inheritance.ˮ Chapter. Four, “Dominance and mutilation in embryo and larvae.ˮ Unpublished MS, Pearson/5/2/10/4, Karl Pearson Papers, University College London Archives, London.

7 For another discussion on this aspect of Jenkinson’s paper, see Sapp (1987).

8 Huxley. “Notes on J. W. Jenkinson Embryol. Lectures 1907 or 1908.ˮ Box 2, Folder 3, 1907-1908, Huxley Papers.

9 Huxley. Manuscript titled “The Functions of the Brainˮ dated to 1907. Box 2, Folder 2, Huxley Papers.

10 Huxley. Manuscript titled “Natural Selectionˮ dated to 1907. Box 2, Folder 2, Huxley Papers.

11 Huxley. Manuscript titled “The Functions of the Brainˮ dated to 1907. Box 2, Folder 2, Huxley Papers.

12 Huxley. Manuscript titled “The Functions of the Brainˮ dated to 1907. Box 2, Folder 2, Huxley Papers.

13 Huxley. Manuscript titled “The Functions of the Brainˮ dated to 1907. Box 2, Folder 2, Huxley Papers.

14 Huxley. Manuscript titled “The Functions of the Brainˮ dated to 1907. Box 2, Folder 2, Huxley Papers.

15 Huxley. Manuscript titled “The Functions of the Brainˮ dated to 1907. Box 2, Folder 2, Huxley Papers.

16 Huxley. Manuscript titled “The Functions of the Brainˮ dated to 1907. Box 2, Folder 2, Huxley Papers.

17 Huxley. Manuscript titled “The Functions of the Brainˮ dated to 1907. Box 2, Folder 2, Huxley Papers.

18 Huxley. Notebook entry dated Oct 8th Tuesday. Box 2, Folder 3, Huxley Papers.

19 Huxley. Essay titled “Natural Selection & Lamarckismˮ dated 1908. Box 2, Folder 4, Huxley Papers.

20 Huxley. Essay titled “Natural Selection & Lamarckismˮ dated 1908. Box 2, Folder 4, Huxley Papers.

21 W. F. R. Weldon upheld a similar view. See also Radick (2005).

22 Huxley. Notebook titled “Mar-Apr. 1909ˮ. Box 2, Folder 6, Huxley Papers.

23 I am here using the copy of Jenkinson’s book located at the Woodson Research Centre, Fondren Library, Rice University (Reference No. QL955 .J5). John W. Jenkinson. 1909. *Experimental Embryology*. Oxford: Clarendon Press, p. 14.

24 For the subsequent interaction between Huxley and Conklin, see Sommer (2014).

25 For more on the discordances between the grandfather and grandson on this topic, see Lidgard and Nyhart (2017).

26 Conklin held a similar view. For more on this, see Sommer (2014).

27 Huxley. Lecture titled “Biology and War,ˮdated 1916. Quotation on p. 44. Box 57, Folder 6, Huxley papers.

28 Huxley. Lecture titled “Biology and War,ˮ dated 1916. Quotation on p. 40. Box 57, Folder 6, Huxley Papers.

29 Huxley. Lecture titled “Biology and War,ˮ dated 1916. Quotation on p. 40. Box 57, Folder 6, Huxley Papers.

30 Huxley to William Bateson, June 25, 1925. Box H3, Letter Ref. Number 1902, The Bateson Letters Collection, John Innes Centre, Norwich, UK.

31 Huxley to William Bateson, December 14, 1925. Letter Ref. Number G2K18A, The Bateson Letters Collection, John Innes Centre, Norwich, UK.

32 Huxley to Lillie, March 31, 1928. Box 5, Folder 47, Frank Rattray Lillie Papers, Marine Biological Laboratory Archives, Woods Hole, Massachusetts.

## References

[CR1] Allen, Garland E. 1975. *Life science in the twentieth century*. New York: Wiley.

[CR2] Allen, Garland E. 1992. Julian Huxley and the eugenics view of human evolution. In *Julian Huxley: Biologist and statesman of science*, ed. C. K. Waters and A. Van Helden, 193–222. Houston: Rice University Press.

[CR3] Anon. 1916. Geoffrey Watkins Smith. *Nature* 97:502.

[CR4] Baedke, Jan. 2024. Evolution and development. *Stanford Encyclopedia of Philosophy,* https://plato.stanford.edu/entries/evolution-development/ [Accessed 1 Mar 2025].

[CR5] Bartley, Mary M. 1995. Courtship and continued progress: Julian Huxley’s studies on bird behavior. *Journal of the History of Biology* 28:91–108.

[CR6] Bashford, Alison. 2022. *The Huxleys: An intimate history*. Chicago: Chicago University Press.

[CR7] Blacker, Carlos P. 1967. Obituary: [Sir Alexander Carr-Saunders] 14th January 1886–6th October 1966. *Population Studies* 20:365–369. 22084950 10.1080/00324728.1967.10409969

[CR8] Bowler, Peter J. 1983. *The eclipse of Darwinism: Anti-Darwinian evolution theories in the decades around 1900*. London: John Hopkins University Press.

[CR9] Bowler, Peter J. 1989. *The Mendelian revolution: The emergence of hereditarian concepts in modern science and society*. London: Athlone Press. 10.1126/science.247.4940.348 17735857

[CR10] Brooks, Ross. 2021. *Weird sex science in Britain ca. 1900–1939: Narratives of naturalisation and eradication*. PhD diss., Oxford Brookes University.

[CR11] Brooks, Ross. 2023. Mendel’s closet: Genetics, eugenics, and the exceptions of sex in Edwardian Britain. *Notes and Records*:1–36.

[CR12] Cain, Joe. 2010. Julian Huxley, general biology and the London Zoo, 1935–42. *Notes and Records of the Royal Society* 64:359–378.

[CR13] Carr-Saunders, Alexander. 1922. *The population problem: Study of human evolution*. Oxford: Clarendon Press.

[CR14] Churchill, Frederick B. 1992. *The elements of experimental embryology*: A synthesis for animal development. In *Julian Huxley: Biologist and statesman of science*, ed. C. K. Waters and A. Van Helden, 107–126. Houston: Rice University Press.

[CR15] Churchill, Frederick B. 2007. Living with the biogenetic law: A reappraisal. In *From embryology to evo-devo: A history of developmental evolution*, ed. Manfred D. Laubichler and Jane Maienschein, 37–81. Cambridge, MA: The MIT Press.

[CR16] Churchill, Frederick B. 2015. *August Weismann: Development, heredity, and evolution*. Cambridge, MA: Harvard University Press.

[CR17] Deichmann, Ute. 2016. Epigenetics: The origins and evolution of a fashionable topic. *Developmental Biology* 416:249–254. 27291929 10.1016/j.ydbio.2016.06.005

[CR18] Erlingsson, Steindór J. 2013. Institutions and innovation: Experimental zoology and the creation of the British Journal of Experimental Biology and the Society for Experimental Biology. *British Journal for the History of Science* 46:73–95.

[CR19] Esposito, Maurizio. 2013. *Romantic biology, 1890–1945*. London: Routledge.

[CR20] Ford, Edmund B. 1931. *Mendelism and evolution*. London: Methuen & Co.

[CR21] Geison, Gerald. 1978. *Michael Foster and the Cambridge school of physiology: The scientific enterprise in late Victorian society*. Princeton, NJ: Princeton University Press.

[CR22] Gould, J. Stephen. 1977. *Ontogeny and phylogeny*. Cambridge, MA: The Belknap Press of Harvard University Press.

[CR23] Hodge, J. M. S. 1990. Review of Peter J. Bowler *The non-Darwinian revolution: Reinterpreting a historical myth*. *British Journal for the History of Science* 23:332–334.

[CR24] Horder, J. Tim. 2008. A history of evo-devo in Britain: Theoretical ideals confront ideological complexity. *Annals of the History and Philosophy of Biology* 40:101–174.

[CR25] Foster, Michael, and E. Ray Lankester, eds. 1898. *The scientific memoirs of Thomas Henry Huxley*. London: Macmillan and Co., Limited.

[CR26] Huxley, Julian S. 1912. *The individual in the animal kingdom*. Cambridge: Cambridge University Press.

[CR27] Huxley, Julian S. 1921. Some implications of the chromosome theory of heredity. *Science Progress in the Twentieth Century (1919–1933)* 16:235–250.

[CR28] Huxley, Julian S. 1923. The physiology of sex-determination. *Nature* 112:927–930.

[CR29] Huxley, Julian S., and A. Carr-Saunders. 1924. Absence of prenatal effects of lens-antibodies in rabbits. *British Journal of Experimental Biology* 1:215–248.

[CR30] Huxley, Julian S. and E. B. Ford. 1925. Mendelian genes and rates of development. *Nature* 116:861–863.

[CR31] Huxley, Julian S. 1926. Review: A static theory of heredity. *The Quarterly Review of Biology* 1:578–584.

[CR32] Huxley, Julian S. and G. De Beer. 1934. *The elements of experimental embryology*. Cambridge: Cambridge University Press.

[CR33] Huxley, Julian S. 1936a. Eugenics and society. *Eugenics Review* 28:11–31. 21260194 PMC2985562

[CR34] Huxley, Julian S. 1936b. Natural selection and evolutionary progress. *Report of BAAS annual meeting 9-16 September*, Section D-Zoology:81-100.

[CR35] Huxley, Julian S. 1942. *Evolution: The modern synthesis*. London: Allen & Unwin.

[CR36] Huxley, Julian S. 1958. *Biological aspects of cancer*. New York: Harcourt, Brace and Company.

[CR37] Huxley, Julian S. 1970. *Memories*. London: Harper & Row, Publishers.

[CR38] Huxley, Julian S., Massimo Piggliuci, and Gerd Müller. 2009. *Evolution: The modern synthesis – the definitive edition*. Cambridge, MA: The MIT Press.

[CR39] Jenkinson, John W. 1906. Remarks on the germinal layers of vertebrates and on the significance of germinal layers in general. *Manchester Literary & Philosophical Society (Manchester Memoirs)* 50:1–89.

[CR41] Lamm, Ehud. 2011. Review of Julian Huxley, *Evolution: The modern synthesis – the definitive edition*, with a new foreword by Massimo Pigliucci and Gerd B. Müller. *Integrative Psychological and Behavioral Science* 45:154–159.

[CR42] Lidgard, Scott, and K. Lynn Nyhart. 2017. *Biological individuality: Integrating scientific, philosophical, and historical perspectives*. Chicago: Chicago University Press.

[CR43] Loison, Laurent. 2022. The environment: An ambiguous concept in Waddington’s biology. *Studies in History and Philosophy of Science* 91:181–190. 34952238 10.1016/j.shpsa.2021.12.003

[CR44] Maienschein, Jane. 1991. The origins of Entwicklungsmechanik. In *A conceptual history of modern embryology*, ed. Scott Gilbert. *Developmental Biology* 7:35–67. Boston, MA: Springer.

[CR45] Maienschein, Jane. 2007. The cell as the basis for heredity, development, and evolution: Richard Goldschmidt’s program of physiological genetics. In *From embryology to evo-devo: A history of developmental evolution*, ed. Manfred Laubichler and Jane Maienschein, 169–211. Cambridge, MA: MIT Press.

[CR76] Marett, Robert R. 1917. Biographical note to John W. Jenkinson's *Three lectures on experimental embryology, xi-xvi*. Oxford: Clarendon Press.

[CR46] Medawar, Peter. 1982. *Pluto’s republic: Incorporating the art of the soluble and induction and intuition in scientific thought*. Oxford: Oxford University Press.

[CR47] Meulendijks, Max. 2021. Eclipsing the eclipse? A neo-Darwinian historiography revisited. *Journal of the History of Biology* 54:403–443. 34499295 10.1007/s10739-021-09650-9

[CR48] Müller-Wille, Staffan, and Hans-Jörg. Rheinberger. 2012. *A cultural history of heredity*. Chicago: Chicago University Press.

[CR49] Nicoglou, Antonine. 2024. *Plasticity in the life sciences*. Chicago: The University of Chicago Press.

[CR50] Olby, Robert. 1992. Huxley’s place in twentieth-century biology. In *Julian Huxley: Biologist and statesman of science*, ed. C. K. Waters and A. Van Helden, 53–78. Houston: Rice University Press.

[CR51] Peterson, Erik. 2016. *The life organic: The theoretical biology club and the roots of epigenetics*. Pittsburgh: The University of Pittsburgh Press.

[CR52] Poulton, Edward B. 1908. Theories of heredity. In *Essays of evolution 1889–1907*, ed. E. B. Poulton, 120–138. Oxford: Clarendon Press.

[CR53] Provine, William B. 1971. *The origins of theoretical population genetics*. Chicago: The University of Chicago Press.

[CR54] Radick, Gregory. 2005. Other histories, other biologies. *Royal Institute of Philosophy Supplement* 56:3–4.

[CR55] Radick, Gregory. 2017. Animal agency in the age of the modern synthesis: W. H. Thorpe’s example. *BJHS Themes* 2:35–56.

[CR56] Radick, Gregory. 2023. *Disputed inheritance: The battle over Mendel and the future of biology*. Chicago: Chicago University Press.

[CR57] Radick, Gregory. 2024. The Baldwin Effect and the potentialities for thoughtful Darwinism around 1900. In *The riddle of organismal agency: New historical and philosophical reflections*, ed. A. Fabregas-Tejeda, Jan Baedke, G. Prieto, and Gregory Radick, 97-113. New York: Routledge.

[CR58] Richmond, Marsha L. 2000. T. H. Huxley’s criticism of German cell theory: An epigenetic and physiological interpretation of cell structure. *Journal of the History of Biology* 33:247–289. 11640226 10.1023/a:1004881730937

[CR59] Richmond, Marsha L. 2009. The cell as the basis for heredity, development, and evolution: Richard Goldschmidt’s program of physiological genetics. In *From embryology to evo-Devo: a history of developmental evolution*, ed. Manfred D. Laublicher and Jane Maienschein, 169–211. Cambridge, MA: The MIT Press.

[CR60] Ridley, Mark. 1986. Embryology and classical zoology in Great Britain. In *A history of embryology*, ed. T. J. Holder, A. Jan Witkowski, and C. C. Wylie, 35–67. Cambridge: Cambridge University Press.

[CR61] Sapp, Jan. 1987. *Beyond the gene: Cytoplasmic inheritance and the struggle for authority in genetics*. Oxford: Oxford University Press.

[CR62] Shan, Yafeng. 2020. *Doing integrated history and philosophy of science: A case study of the origin of genetics*. Cham: Springer.

[CR63] Smith, Geoffrey W. 1906. *Rhizocephala*. Berlin: R. Friedländer & Sohn.

[CR64] Smocovitis, Vassiliki B. 1996. *Unifying biology: The evolutionary synthesis and evolutionary biology*. Princeton: Princeton University Press. 10.1007/BF01947504 11623198

[CR65] Smocovitis, Vassiliki B. 2019. Historicizing the synthesis: Critical insights and pivotal moments in the long history of evolutionary theory. In *The theory of evolution: Principles, concepts, and assumptions*, ed. Samuel M. Scheiner and David P. Mindell, 25–45. Chicago: University of Chicago Press.

[CR66] Smocovitis, Vassiliki B. 2023. Every evolutionist their own historian: The importance of history, context, and the extended evolutionary synthesis. In *Evolutionary biology: Contemporary and historical reflections upon core theory*, ed. Dickins, Thomas. E. and Benjamin J. A. Dickins 25-54. Cham: Springer.

[CR67] Sommer, M. 2014. Biology as technology of social justice in interwar Britain: Arguments from evolutionary history, heredity, and human diversity. *Science, Technology, & Human Values* 39:561–586.

[CR68] Sturdy, W. Steven. 1987. *A co-ordinated whole: The life and work of John Scott Haldane*. PhD diss., The University of Edinburgh.

[CR69] Swiatczak, Bartlomiej. 2023. Evolution within the body: The rise and fall of somatic Darwinism in the late nineteenth century. *History and Philosophy of the Life Sciences* 45:1–27. 36862350 10.1007/s40656-023-00566-7

[CR70] Tabery, James. 2014. *Beyond versus: The struggle to understand the interaction of nature and nurture*. Cambridge, MA: The MIT Press.

[CR71] Walsh, Denis. 2015. *Organisms, agency, and evolution*. Cambridge: Cambridge University Press.

[CR72] Waters, C. Kenneth, and A. Van Helden, ed. 1992. *Julian Huxley: Biologist and statesman of science*. Houston: Rice University Press.

[CR73] Weldon, Walter R. F. 1883. Note on the early development of *Lacerta muralis*. *Quarterly Journal of Microscopical Science* 2:134–144.

[CR74] Wells, H. G., Julian S. Huxley, and P. G. Wells. 1931. *The science of life*. London: Cassell and Company Limited.

[CR75] Witkowski, A. Jan. 1992. Julian Huxley in the laboratory: Embracing inquisitiveness and widespread curiosity. In *Julian Huxley: Biologist and statesman of science*, ed. C. K. Waters and A. Van Helden, 79–103. Houston: Rice University Press.

